# Serum miR-34a as Indicator of Impaired Fibrinolytic Capacity in Pediatric Thrombosis Through Inadequate Regulation of the ACE/PAI-1 Axis

**DOI:** 10.3390/ijms262010110

**Published:** 2025-10-17

**Authors:** Iphigenia Gintoni, Kleoniki Baldouni, Athina Dettoraki, Aikaterini Michalopoulou, Ioanna Papathanasiou, Aspasia Tsezou, Dimitrios Vlachakis, Helen Pergantou, George P. Chrousos, Christos Yapijakis

**Affiliations:** 1 Unit of Orofacial Genetics, 1st Department of Pediatrics, School of Medicine, National Kapodistrian University of Athens, “Aghia Sophia” Children’s Hospital, 115 27 Athens, Greece; iph.gintoni@gmail.com (I.G.); kleoniki_km@hotmail.com (K.B.); 2 University Research Institute of Maternal and Child Health and Precision Medicine, National Kapodistrian University of Athens, Choremion Laboratory, “Aghia Sophia” Children’s Hospital, 115 27 Athens, Greece; chrousge@med.uoa.gr; 3 Haemophilia Center for Children and Adolescents, Haemostasis and Thrombosis Unit, “Aghia Sophia” Children’s Hospital, 115 27 Athens, Greece; athinadett@gmail.com (A.D.); katia.michalopoulou@gmail.com (A.M.); hpergantou@gmail.com (H.P.); 4 Laboratory of Cytogenetics and Molecular Genetics, Faculty of Medicine, School of Health Sciences, University of Thessaly, Biopolis, 41500 Larissa, Greece; iopapat@uth.gr (I.P.); atsezou@uth.gr (A.T.); 5 Laboratory of Genetics, Department of Biotechnology, School of Applied Biology and Biotechnology, Agricultural University of Athens, 11855 Athens, Greece; dimvl@aua.gr

**Keywords:** thrombosis in childhood, adolescent thrombosis, fibrinolysis, coagulation, thrombophilia, stroke, microRNA, miR-34a-5p, miR-145-5p, epigenetics, SERPINE1 4G/5G polymorphism, ACE I/D polymorphism

## Abstract

Pediatric thrombosis (PT) represents a rare condition that can manifest from neonatal life to adolescence, encompassing life-threatening complications. Its pathogenesis is attributed to immature hemostasis in conjunction with environmental and genetic factors, predominantly including those resulting in increased levels of plasminogen activator inhibitor 1 (PAI-1), the principal inhibitor of fibrinolysis, which is subject to upstream regulation by angiotensin-converting enzyme (ACE). Although the implication of microRNAs (miRNAs), epigenetic modulators of gene expression, has been demonstrated in adult thrombosis, evidence is lacking in the pediatric setting. Here, we investigated the involvement of two miRNA regulators of PAI-1 (*SERPINE1* gene) in PT, in relation to clinical and genetic parameters that induce PAI-1 fluctuations. Following bioinformatic target-prediction, miRNA expression was assessed by quantitative real-time PCR in serum-samples of 19 pediatric patients with thrombosis (1–18 months post-incident), and 19 healthy controls. Patients were genotyped for the *SERPINE1*-4G/5G and *ACE*-I/D polymorphisms by PCR-based assays. Genotypic and thrombosis-related clinical data were analyzed in relation to miRNA-expression. Two miRNAs (miR-145-5p, miR-34a-5p) were identified to target *SERPINE1* mRNA, with miR-34a additionally targeting the mRNA of *ACE*. The expression of miR-34a was significantly decreased in patients compared to controls (*p* = 0.029), while no difference was observed in miR-145 expression. Within patients, miR-34a expression demonstrated a peak 1–3 months post-thrombosis and was diminished upon treatment completion (*p* = 0.031), followed by a slight long-term increase. MiR-34a levels differed significantly by thrombosis site (*p* = 0.019), while a significant synergistic interaction between site and onset type (provoked/unprovoked) was detected (*p* = 0.016). Finally, an epistatic modification was observed in cerebral cases, since double homozygosity (4G/4G + D/D) led to a miR-34 decrease, with D/D carriership reversing the 4G/4G-induced upregulation of miR-34a (*p* = 0.006). Our findings suggest that in pediatric thrombosis, downregulation of miR-34a is indicative of impaired fibrinolytic capacity, attributed to deficient regulation of the inhibitory ACE/PAI-1 axis.

## 1. Introduction

Thrombosis is characterized by abnormal clot formation within a blood vessel, obstructing blood flow and resulting from a substantial imbalance between coagulation and fibrinolysis. The developed thrombotic complexes mostly comprise red blood cells, fibrin, as well as platelets and leukocytes [1,2]. Although conditions such as an ischemic stroke (IS) or pulmonary embolism (PE) caused by thrombus development are typically seen in adults, thrombosis is also a serious health complication of pediatric populations and can be classified as venous or arterial, depending on the affected vessels, with venous thromboembolism (VTE) scoring the highest rates [3,4,5].

While pediatric thrombosis is considered rare, corresponding to up to 14/10,000 annual admissions, its incidence rates have demonstrated a significant increase by 70%, with a 7-fold increase only corresponding to strokes affecting newborns and children, with newborns significantly higher thrombotic rates, due to unstable hemostasis [1,4,5,6,7]. Thrombotic incidents in pediatric patients most frequently present as cerebral thrombosis, followed by limb incidents and entail life-threatening complications, such as pulmonary embolism and post-thrombotic syndrome [4,8,9]. Early detection and thorough anticoagulant/thrombolytic treatment and subsequent chemoprophylaxis can be determinant to patients’ survival, complication occurrence, and overall therapeutic outcomes, while thorough and multifactorial evaluation of a child’s risk is crucial for their clinical management as to prevention of secondary thrombosis, post-thrombotic syndrome, and overall recurrence [4,6,8,10].

Although pediatric thrombosis is multifactorial and its manifestation is affected by possible risk factors such as hypoxia, inflammation-involving conditions, malignancy, and injury, it demonstrates a strong and well-established genetic background that increases a child’s vulnerability to thrombi formation [9,11,12,13]. Inherited thrombophilia is considered a solid risk factor in pediatric thrombosis and includes the presence of genetic variants causing functional or quantitative imbalances in factors involved in hemostasis and fibrinolysis, such as factor V (FV) Leiden, prothrombin (PTH), methylenetetrahydrofolate reductase (MTHFR), plasminogen activator inhibitor-1 (PAI-1), and angiotensin-converting enzyme (ACE) [11,14]. Amongst the above, PAI-1 has recently been reported as the predominant inherited genetic risk factor for pediatric thrombosis [9].

PAI-1 is the most robust inhibitor of fibrinolysis, a process that acts at the final hemostatic stage by dissolving developed thrombi, through the enzymatic activity of plasmin, thus restoring normal blood circulation [13,15,16,17]. PAI-1, which is encoded by the *SERPINE1* gene, is predominantly secreted by endothelial cells and negatively regulates the fibrinolytic system mainly by its covalent binding to tissue plasminogen activator (tPA), obstructing the conversion of plasminogen to plasmin, which ultimately catalyzes the degradation of fibrin, the main stabilizer of blood clots [2,13,18,19]. The ratio of tPA/PAI-1 is unfavorable for effective fibrinolysis during childhood, since tPA levels are significantly reduced at birth and until late adolescence, compared to the increased levels of its primary inhibitor, PAI-1 [19].

It has been demonstrated that increased PAI-1 levels ultimately lead to diminished fibrinolytic activity and a hypercoagulable state, therefore skyrocketing the risk of both venous and arterial thrombosis, while the generation of thrombin itself acts as an inducer of additional PAI-1 secretion [13,15,16,17,20]. It is experimentally supported that upregulated PAI-1 levels not only promote thrombotic development but may also contribute to resistance against thrombolytics [19,21]. *SERPINE1* expression has been demonstrated to increase prior to thrombus development, while maximum PAI-1 levels are reached during the acute phase of thrombosis and finally decrease significantly post-treatment, which includes tPA in life-threatening incidents [5,15,22,23]. However, following a thrombotic incident, PAI-1 has been shown to remain increased for prolonged periods of time, with a minimum of three months, contributing to proportional PAI-1/t-PA abnormalities and therefore decreased fibrinolytic capacity in up to 54% patients [24].

PAI-1 levels are highly influenced by the 4G/5G (rs1799768) 1bp insertion/deletion functional polymorphism in the promoter region of the *SERPINE1* coding gene [15,25,26]. The presence of the 4G polymorphic allele is strongly associated with higher levels of circulating PAI-1, resulting in hypofibrinolysis and coagulation enhancement, while the normal 5G allele has been shown to exert a protective effect against thrombotic recurrence [25,26]. The *PAI*-*1* 4G/5G polymorphism is reported to be the most frequently detected genetic variant within children who have undergone thrombosis, compared to other predisposing factors [9]. Moreover, PAI-1 levels are also regulated by the renin-angiotensin system (RAS), which indirectly suppresses fibrinolysis via angiotensin II (angII) that triggers a significant PAI-1 increase [27,28,29]. In this manner, the angiotensin-converting enzyme (ACE), which is primarily responsible for direct angII formation, serves as an upstream positive regulator of PAI-1, thus exerting a negative effect on fibrinolytic activity.

The insertion/deletion (I/D) functional polymorphism (rs1799752) of the *ACE* gene is widely known for increasing circulating ACE levels in the presence of the D (deletion) allele (II < ID < DD), with the DD genotype associated with maximum concentrations and enzymatic activity for angII formation [30,31]. The presence of the D allele and primarily DD homozygosity has also been demonstrated to indirectly increase PAI-1 levels, resulting in fibrinolytic impairment through the functional ACE/PAI-1 axis [32]

In addition to key proteinic regulators of thrombosis, such as PAI-1, evidence has been mounting around the implication of microRNAs (miRNAs) in the delicate regulatory interplays of adult thrombosis [33]. MiRNAs are a class of single-stranded, small (18–25 nt), non-coding RNA molecules that act as profound negative regulators of gene expression at a post-transcriptional level, through the translational inhibition of mRNA targets. Each miRNA retains the capacity to regulate the expression of multiple gene targets at once, through the binding of its seed sequence to recognized complementary binding sites within the 3′ untranslated region (3′-UTR) of their mRNAs [33,34,35]. Even after their extracellular release, miRNAs remain remarkably stable, due to their binding with Argonaute proteins and/or their encapsulation in extracellular vesicles (EVs), which provide protection against RNase-mediated degradation [36]. Hence, given that their expressional fluctuations (up- or downregulation) can be highly reflective of a pathology, they hold great promise both as biomarkers but also as crucial elements of disease pathogenicity by the abnormal regulation of the implicated genes [34,37].

Despite the increasing evidence on miRNA implication in adult thrombosis, corresponding studies are completely lacking in the pediatric setting. Pediatric thrombosis exhibits a characteristic pathophysiology due to the central role of hypofibrinolysis in light of a child’s developing hemostasis, but also due to the differential impact of genetic predisposition, as well as the lack of accumulation of environmental and age-related risk factors that govern the adult form. Therefore, direct extrapolation of miRNA data from adult studies carries a substantial risk of unreliability, highlighting the need for research specifically tailored to the pediatric context of the pathology.

In the present study, we have examined the expression levels of two miRNA regulators of the ACE/PAI-1 axis (miR-145-5p and miR-34a-5p) in pediatric patients with thrombosis, 1–18 months post-incident, compared to age- and gender-matched healthy controls. Expressional patterns of the studied miRNAs were furtherly evaluated within distinct subpopulations of the patient cohort, in relation to clinical and genetic factors that have been demonstrated to induce anticipated changes in PAI-1 levels. Those variables included post-thrombotic time, thrombosis site, onset type, treatment status, as well as genotypic data for the *SERPINE1*-4G/5G and *ACE*-I/D functional polymorphisms.

This study in pediatric patients was designed under the guiding hypothesis of a possible regulatory interplay between miR-145 and miR-34a expression and PAI-1 fluctuations, which are documented in the literature for hypofibrinolysis in adult thrombosis [5,15,22,23], across different stages of post-thrombotic course and treatment, as well as the different genotypes of the 4G/5G and I/D functional variants. The present research is, to our knowledge, the first globally to investigate miRNA expression profiles in the context of pediatric thrombosis. Its aim is to address the lack of evidence in the genetic/epigenetic interplays that might govern this rare and severe pediatric clinical entity, but also to potentially shed light on aspects of adult thrombosis, where impaired fibrinolysis might not be the central mechanism, but definitely represents an integral component.

## 2. Results

### 2.1. Group Characteristics

A total of 38 participants were included in the present study, including 19 pediatric patients who had undergone thrombosis 1–18 months ago and 19 healthy controls. Summarized in Table 1 are the characteristics of both studied groups (age and gender), as well as the relevant clinical data of the studied patients, including type (AT or DVT) and classification of thrombotic incident (provoked/unprovoked), site of thrombosis, time intervals between thrombosis and sample collection, treatment status, and finally genotypic data on the 4G/5G polymorphism of the *PAI*-*1* (*SERPINE1*) gene, as well as the *ACE* I/D polymorphism. The Mann–Whitney U test was applied to compare the quantitative variable of age, yielding no statistically significant difference between the two groups (*p* = 0.124). Gender-wise, a Chi-Squared Test was performed and did not reveal a statistically significant difference (*p* = 0.163). Regarding the type of presented thrombosis, VT represented most cases, while only 5% corresponded to AT, consistent with epidemiological data for pediatric thrombosis in the general population, as stated in the literature [3,4,5]. Nearly 50% of the enrolled cases had developed cerebral thrombosis, followed by thrombosis of the lower limbs in 30% of patients and pulmonary thromboembolism (PE) in around 20%. It was estimated that 68% of the studied thrombotic incidents were unprovoked, while 32% were injury-induced (provoked).

A positive family history for hereditary thrombosis was reported for 63% of patients, which was defined by the occurrence of at least one affected individual per generation (across four generations) through a single parental line in vertical inheritance, or at least two affected individuals when both the paternal and maternal lineages are involved. As to *PAI*-*1* genotyping for the 4G/5G promoter functional polymorphism, 84% of patients were carriers of at least one 4G allele, which is associated with higher *SERPINE1* expression, increased PAI-1 levels, and risk of thrombosis [8,14,24,25], while only around 16% carried the normal 5G allele in a homozygous state. In relation to the *ACE*-I/D functional polymorphism, around 95% of patients carried at least one D allele, associated with elevated ACE levels, while homozygotes for the normal I allele only accounted for 5% of the patient population (Table 1).

### 2.2. Bioinformatic Analyses of Target Prediction

**Figure 1:**
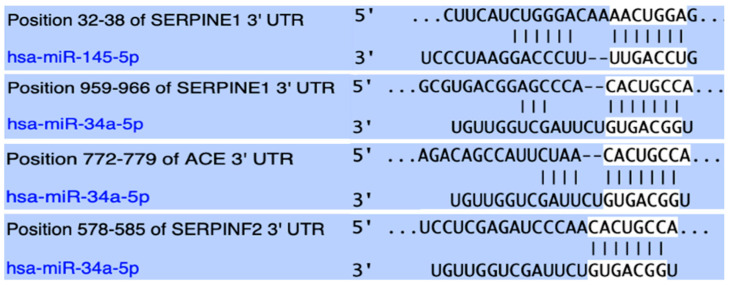
Summary of the predicted miRNA/mRNA targeting as obtained from the “TargetScanHuman” bioinformatic tool. For the total of interactions between the studied miRNAs and the mRNAs of the genes of interest, evolutionary conserved targeting was identified. Presented in the figure are the seed sequences of miR-145 and miR-34a, as well as the binding sites within the 3′-UTRs of the target mRNAs. Complementarity is perfect (7/7) for all complexes and is furtherly supported by the presence of an additional adenin, opposite to the uracil in position 1 of the miRNA, except from the interaction of miR-145 and the mRNA of *SERPINE1*.

Target prediction analyses suggested that both miR-145-5p and miR-34a-5p hold conserved binding sites on positions 32–38 and 959–966 of the 3′-UTR of *SERPINE1* mRNA, respectively. Both score perfect complementarity of the miRNA seed region, while in the case of miR-34a, the mRNA binding site retains an adenine (A), opposite to miRNA position 1 of the seed sequence, thus additionally enhancing the stability of the predicted miRNA/mRNA complex (Figure 1). Both miRNAs demonstrated high microT-scores (>0.7), supporting the validity of predictions. Experimentally validated negative regulation of *SERPINE1* by both miR-145 and miR-34a has been demonstrated through 5 and 21 experiments, respectively, according to DIANA-microT-CDS analysis, including both direct and indirect validation in various cell types, including endothelial cells in the case of miR-34a.

In addition, target prediction was performed to investigate the possible complementarity and regulatory relationship between the above-mentioned miRNAs and the mRNA of the *ACE* gene, which acts as the indirect controller of PAI-1 levels. The analysis yielded positive results only for miR-34a, which holds a binding site at positions 772–779 of *ACE* 3′ UTR, demonstrating perfect 7/7 complementarity and an additional A opposite to position 1 U of the seed sequence (Figure 1), which further stabilizes the miRNA/mRNA complex. The miR-34a/*ACE* regulatory relationship has been validated in eight direct experiments, and it presents with a microT score of 0.78.

Finally, a strong interaction between miR-34a and the mRNA of the *SERPINF2* gene that codes for α2-antiplasmin, the main inactivator of fibrinolytic plasmin, was predicted. The binding site for miR-34a in the 3′-UTR of *SERPINF2* is documented at position 578–585, providing perfect complementarity with the seed sequence and an additional adenine opposite to the uracil in position 1 of miR-34a (Figure 1), thus enhancing binding stability and possible regulatory efficiency. Although this particular binding site exhibits high evolutionary conservation, experimental validation of the regulatory relationship between miR-34a and the *SERPINF2* gene is not yet available.

### 2.3. MiRNA Expression Profiling Between Patients and Controls

**Figure 2:**
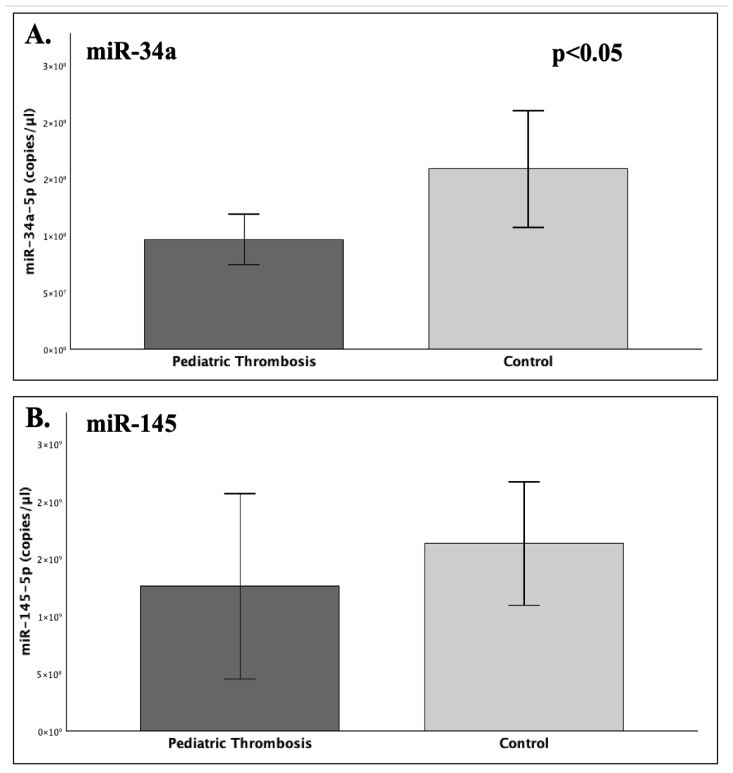
The graphs depict the mean expression (copies/μL) of (**A**) miR-34a-5p and (**B**) miR-145-5p that was measured in serum samples of pediatric patients with thrombosis (1–18 months post-incident) and healthy controls. It was shown that miR-34a was significantly downregulated (*p* = 0.029) in the patient population compared to controls, while the expression of miR-145-5p exhibited no statistical difference between the two groups.

The results of the expressional quantification (copies/μL) of the studied miRNAs between patients and controls revealed that the levels of miR-34a-5p were significantly decreased by around 40% in the group of patients, compared to controls (FC = 0.61; *p* = 0.029). As to the expression of miR-145-5p, a similar trend towards higher levels in the control population was observed, but did not reach statistical significance (*p* = 0.068) (Figure 2).

### 2.4. MiRNA Expression Profiling Within Patients in Relation to Different Factors

#### 2.4.1. Time Post-Thrombosis

**Figure 3:**
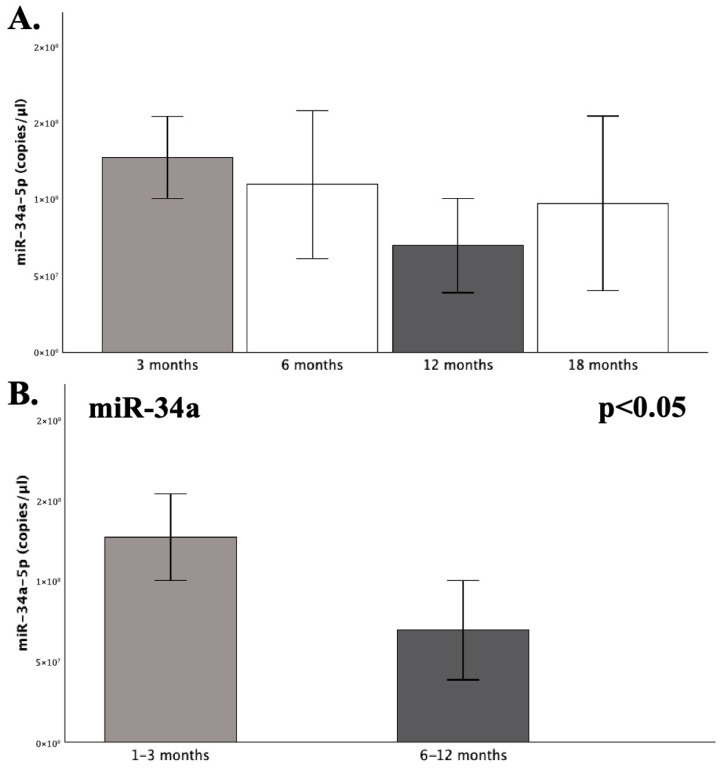
Graphs depicting the mean expression (copies/μL) of miR-34a-5p (**A**) across the four defined groups of post-thrombotic time and (**B**) between the 1–3- and 6–12-month groups, which differed significantly in the expression of miR-34a. Statistical significance was set at *p* < 0.05.

Within the group of patients, to assess whether the levels of the studied miRNAs fluctuate over time following thrombosis, the omnibus tests of one-way ANOVA and Kruskal–Wallis were performed, with respect to the distribution of variables. Additionally, to evaluate monotonic trends across the time groups of patients and subsequent exploratory pairwise comparisons, the Jonckheere–Terpstra (JT) test for ordered variables was applied. The expression levels of miR-145 and miR-34a did not exhibit significant overall significance across time post-thrombosis (*p* > 0.05). However, the 1–3- and 6–12-month time groups differed significantly as to the expression of miR-34a, with the latter exhibiting approximately 45% lower expression compared to the 1–3 group (FC = 0.55; *p* = 0.029) (Figure 3), which was additionally confirmed by the conduction of a *t*-test for independent samples (*p* = 0.031). Pairwise comparisons, by the employment of an independent-sample *t*-test, were also performed for all the other time groups, which further confirmed the lack of significance as to the expression of both miR-145 and miR-34a (*p* > 0.05). Although not statistically significant, miR-34a remains around 39% reduced during the final 12–18 post-thrombotic interval of the patients, compared to its mean expression in the control group (FC = 0.61; *p* = 0.348) (Figure 3).

#### 2.4.2. PAI-1 Genotype

**Figure 4:**
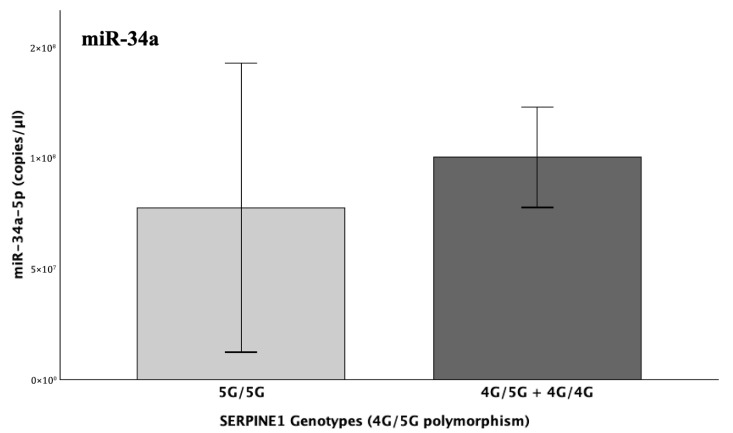
Bars depicting the mean expression (copies/μL) of miR-34a-5p across genotypic groups for the 4G/5G functional polymorphism of the *SERPINE1* gene, which increases the expression of *PAI*-*1* in presence of the 4G allele.

Given the fact that both miR-145 and miR-34a are regulators of *PAI*-*1*, their expression was evaluated across the different genotypic groups among the patients with regard to the gene’s 4G/5G (rs1799768) promoter polymorphism. All performed tests (One-way ANOVA, Kruskal–Wallis and Jonckheere–Terpstra) indicated no statistical significance in the expression of the two miRNAs among the genotypic groups (5G/5G, 4G/5G and 4G/4G) (*p* > 0.05), while additional analysis under a dominant model (carriers versus non-carriers of the 4G allele), also yielded insignificant results (Mann–Whitney U/independent-sample *t*-test, *p* > 0.05). Similarly, pairwise comparisons did not identify any significant differences in miRNA expression among genotypes. Despite the absence of statistical significance, the yielded graphs for miR-34a indicate a mild increase in light of increasing 4G-allele count (Figure 4), while miR-145 did not exhibit any consistent visual trend. This pattern in miR-34a, although highly consistent with biological plausibility and the above findings, did not reach statistical significance, probably due to the markedly imbalanced genotypic distributions, since most of the patients (84%) were carriers of the pathological 4G allele, not allowing for even comparisons across groups.

#### 2.4.3. Treatment Status

Patients were collected in variable timeframes following their thrombotic incidents. Therefore, almost half of the participants (47.4%) within the patient group were under active treatment (including chemoprophylaxis) at the time of sample collection, while the other half (52.6%) were not, either due to recent termination (1–3 months) or long-term discontinuation. Hence, to assess whether the treatment affects the expression of the studied miRNAs, the two groups were compared as to miRNA levels factored by treatment status (on/off). The expression of miR-145-5p did not differ significantly according to therapy status (Mann–Whitney U test, *p* = 0.902), while the respective result for miR-34a was also not significant but exhibited a slightly decreasing trend within the actively treated patients (independent-sample *t*-test, *p* = 0.06). Finally, general linear models were used to evaluate the possible effect of the interaction between time post-thrombosis and treatment status on miRNA expression, which also yielded non-significant results (*p* > 0.05). In our analysis, treatment was grouped under the broader category of pharmacological inhibition of thrombogenesis, since anticoagulant or fibrinolytic treatments ultimately converge on the same downstream biological effect: significant reduction in fibrinolysis inhibitors, either by decreasing thrombin (and subsequently fibrin) formation or by directly enhancing fibrinolysis. In both cases, the pathways of interest are exogenously modulated in a similar manner.

#### 2.4.4. Recent Treatment Completion

**Figure 5:**
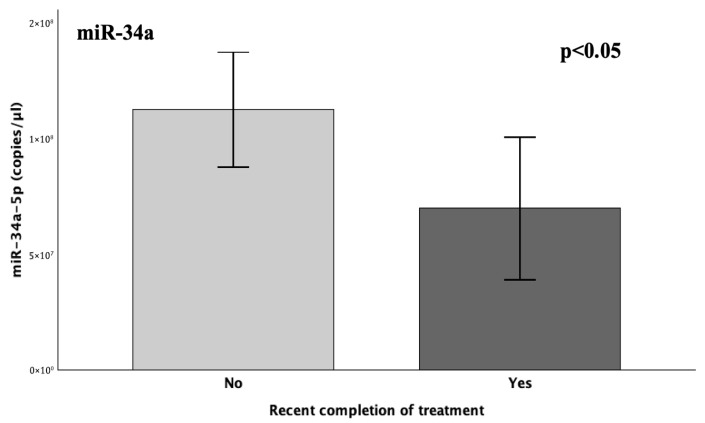
The graphs depict the mean expression (copies/μL) of miR-34a-5p between patients who had recently completed a treatment regimen (1–3 months) and patients either actively receiving or having long-term terminated treatment courses. Statistical significance was set at *p* < 0.05.

As yielded from previous analyses, the lowest miR-34a expression was observed in the 6–12-month group, which aligns with recent therapy completion (1–3 months) for the respective patients, a time point during which the levels of its target (*PAI*-*1*) are diminished, according to literature [5,15,22,23]. Hence, recent treatment completion was also examined as a binary variable in the overall addressing of therapy status for both miRNAs within the patient group (Figure 5). The expression of miR-145 did not exhibit any statistically significant difference between the examined groups, according to the Mann–Whitney U test (*p* = 0.375). On the other hand, miR-34a exhibited a significant 38% downregulation in the group that had recently completed treatment compared to the total of those actively receiving treatment, as well as those who had discontinued treatment for more extended periods and were collected during long-term follow-ups (FC = 0.62; *p* = 0.048) (Figure 1 and Figure 5).

### 2.5. MiR-34a Expression Profiling Within Patients in Relation to Different Factors

As per the yielded significant patterns in the above analyses and overall significance between patients and controls, coupled with supporting bioinformatic evidence indicating strong regulatory relationships with PAI-1 and its positive regulator, ACE, we opted to analyze the expression of miR-34a across additional parameters, in pursuit of a clearer elucidation of its role in pediatric thrombosis.

#### 2.5.1. Family History

Within the patient group, miR-34a levels were assessed in relation to the presence of a thrombosis-positive family history, in accordance with the respective set criteria. In both cases, no statistical significance was yielded by the performed Mann–Whitney U test and independent-sample *T*-test, respectively (*p* > 0.05), thus indicating no effect on miRNA expression.

#### 2.5.2. Anatomical Site and Thrombosis Onset Type

**Figure 6:**
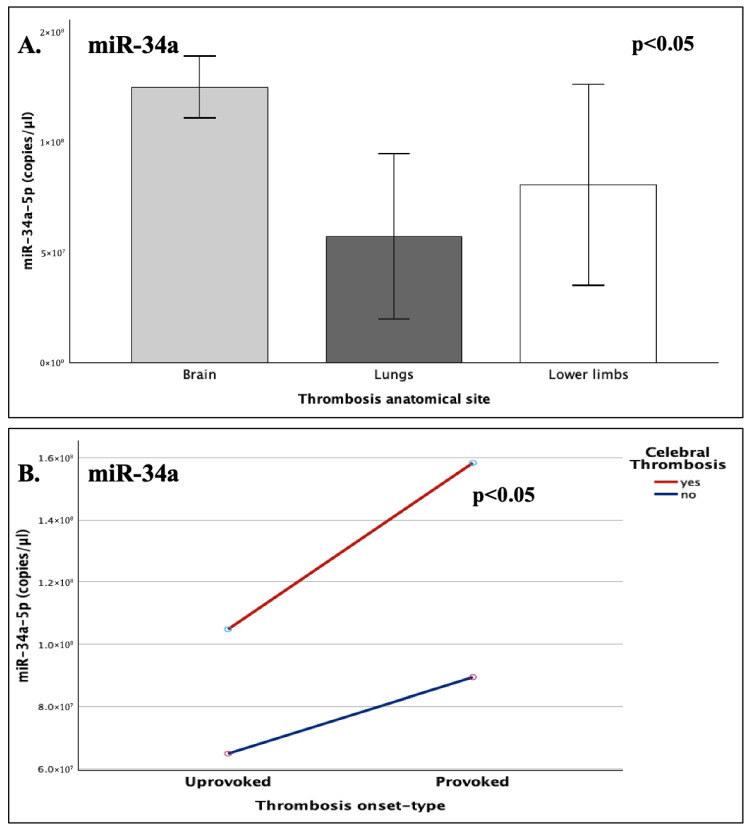
The graphs present the expression (copies/μL) of (**A**), miR-34a-5p across different anatomical sites, where the respective thrombi were developed, including cerebral thrombosis (brain), pulmonary embolism (lung), and lower-limb thrombosis; and (**B**), miR-34a-5p under the combined influence of cerebral involvement and onset-type (provoked/unprovoked).

To address potential site-specific expression patterns of miR-34a, patients were stratified according to the anatomical site of the developed thrombi in three groups: cerebral, pulmonary, and lower-limb thrombosis. An omnibus one-way ANOVA, comparing miR-34a expression across all anatomically defined groups, revealed an overall significant difference (*p* = 0.019), hence underlining a strong correlation between thrombus site and miRNA expression (Figure 6). The most significant disparity was noted between the subgroups of cerebral and pulmonary thrombosis, with cerebral cases demonstrating a 76% increase in miR-34a expression, compared to those of pulmonary thromboembolism (FC = 1.76; *p* = 0.007).

The cerebral subgroup incorporated both unprovoked and injury-following thrombotic incidents, while the majority (75%) of PE cases were unprovoked. Hence, a Mann–Whitney U test was conducted for the evaluation of miR-34a expression in relation to thrombosis onset type (provoked/unprovoked), which alone did not reach statistical significance (*p* = 0.357). However, when a generalized linear model was employed to assess the possible synergistic interaction between anatomical site and thrombosis onset type, a significant effect was detected, with injury-induced cerebral cases demonstrating a 37% increase in miR-34a levels, compared to the respective cerebral unprovoked subgroup (*p* = 0.016; FC = 1.37).

#### 2.5.3. ACE Genotype

**Figure 7:**
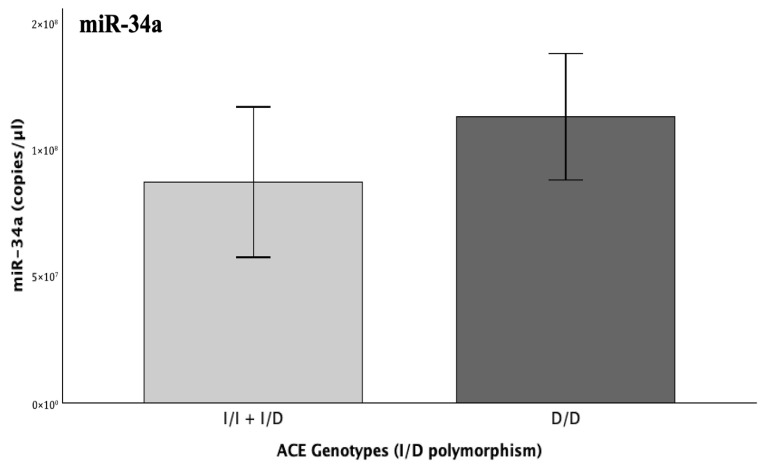
Graph illustrating the expression (copies/μL) of miR-34a-5p across genotypic groups for the I/D functional polymorphism of the *ACE* gene, which increases the levels and enzymatic activity of circulating ACE in presence of the D allele, thus indirectly upstream increasing PAI-1 levels.

Following the genotyping of the patients’ samples for the functional I/D polymorphism of the *ACE* gene, an upstream positive regulator of PAI-1 levels, we opted to evaluate miR-34a overall expression across the three genotypes (II, ID, and DD), which correspond to ascending levels of ACE [28]. The performed one-way ANOVA and JT tests indicated no significant difference across genotypes (*p* > 0.05); however, a clear visual trend for increasing miR-34a expression across the rising count of the ACE-elevating D allele was observed (Figure 7). Since homozygosity for the normal I allele corresponded to only 5% of the studied patients, not adequately allowing for proper omnibus and post hoc comparisons, the comparison between the I/D and D/D was separately evaluated by an independent-sample *T*-test. Indeed, patients carrying the DD genotype presented with 22% increased miR-34a expression, though the results did not reach statistical significance despite the observed trend (FC = 1.22; *p* = 0.355).

#### 2.5.4. Combined Effects of Onset Type, ACE Genotype, and Thrombosis Site

**Figure 8:**
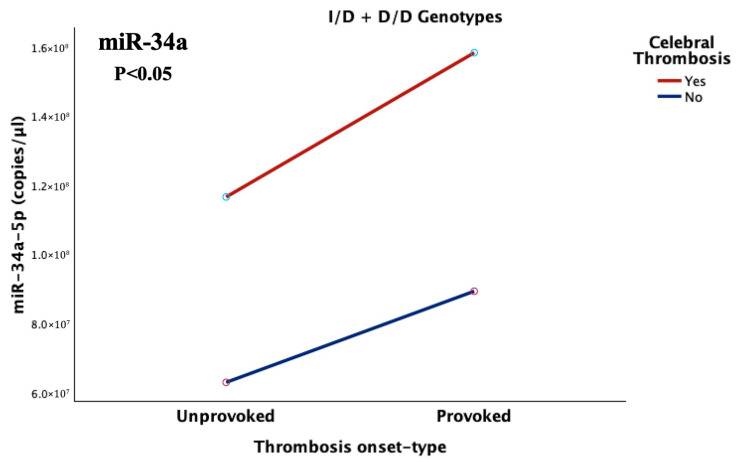
Diagrammatic representation of miR-34a-5p expression under the combined effect of thrombosis site (cerebral/other) and episode onset-type (provoked/unprovoked) in patients carrying at least one D allele for the *ACE*-I/D polymorphism (I/D and D/D genotypes). Trauma-induced (provoked) cerebral cases exhibited significantly elevated miR-34a levels compared to the respective unprovoked cases and to thrombotic episodes of any onset-type in other anatomical locations (*p* = 0.035).

Within the studied patients, a general linear model analysis revealed a significant three-way interaction between thrombosis anatomical site, onset type, and the genotypic presence of at least one D allele of the *ACE* I/D polymorphism (*p* = 0.035), thus underpinning that miR-34a levels were highly influenced by the combined effect of these anatomical, environmental, and genetic variables. In fact, cerebral provoked cases, carrying at least on D allele (I/D and D/D genotypes) demonstrated a 46% increase in miR-34a expression compared to the respective unprovoked ones (FC = 1.46), and a 5.2-fold higher expression (421% increase; FC = 5.22), compared to unprovoked incidents in any other anatomical site (lungs and lower limbs), as illustrated in Figure 8. The generalized linear model (GLM) model explained one third of the observed variance in miR-34a expression, thus indicating a robust, multifactorial effect on miRNA expression (R^2^ = 0.516; adj. R^2^ = 0.330).

#### 2.5.5. Combined Effects of 4G/4G (SERPINE1) and D/D (ACE) Genotypes

A general linear model was also applied to assess the possible combinatorial effect of the 4G/4G and D/D polymorphic genotypes of the *PAI*-*1* and *ACE* genes, which are associated with the maximum levels of the respective encoded factors. Neither 4G nor D homozygosity per se significantly influenced miR-34a expression (*p* = 0.247). However, a parallel univariate GLM analysis including the anatomical site of thrombus development demonstrated a highly significant three-way interaction between *PAI*-*1* 4G homozygosity (4G/4G), *ACE* D homozygosity (D/D), and thrombosis site (cerebral vs. other), which notably affects miRNA expression (*p* = 0.006). Within the patient group, nearly 50% of the expressional heterogeneity of miR-34a was captured by this model (R^2^ = 0.663; adj. R^2^ = 0.494).

**Figure 9:**
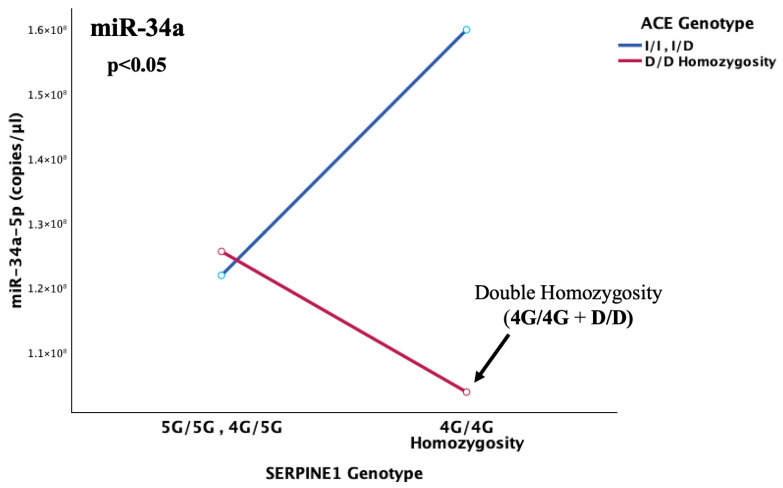
The graph illustrates the epistatic modification, which was observed in cases of cerebral thrombosis, with double homozygosity (4G/4G + D/D) for the *SERPINE1*-4G/5G and *ACE*-I/D gene functional polymorphisms, leading to diminished miR-34a expression, while solely the 4G/4G (in absence of D/D carriership) was associated with the opposite pattern, with miR-34a levels reaching their peak. Hence, the D/D genotype was observed to reverse the significant upregulating effect of 4G/4G homozygosity on miR-34a expression (*p* = 0.006). Both genotypes are associated with the highest genetically predetermined levels of both PAI-1 and ACE in the circulation. The arrow indicates the expression level of the studied miR-34a that corresponds to double homozygosity for the aforementioned variants (4G/4G + D/D).

Interaction plots indicated that among patients who had undergone cerebral thrombosis, the double homozygotes (4G/4G + D/D) exhibited the lowest levels of miR-34a expression. That effect was reversed in 4G/4G carriers in the absence of D/D homozygosity (I/I and I/D genotypes), demonstrating that D/D homozygosity of the *ACE* gene is a critical determinant of whether the 4G allele of *PAI*-*1* positively or negatively affects miR-34a expression (Figure 9).

## 3. Discussion

Childhood thrombosis, including the development of venous and arterial thrombi that obstruct circulation, is a rare condition with its etiological origins lying in the disproportionate imbalance between coagulation and fibrinolytic mechanisms [1,2]. Thrombotic incidents can occur at any stage during a child’s development, from the neonatal period to late adolescence, due to the interplay of environmental risk factors, including hypoxia, inflammation, and injury, alongside a robust genetically predisposing background for thrombophilia [9,11,12,13]. The above risk factors contribute to thrombosis development in a child’s normally immature hemostatic system with a markedly low fibrinolytic capacity, compared to that of adults [1,4,5,6]. Pediatric patients who develop thrombosis come across acute life-threatening or long-term complications, secondary incidents, as well as future thrombotic recurrences, typically managed through anticoagulant/fibrinolytic treatment, chemoprophylaxis, but also by a comprehensive assessment of the children’s overall risk that includes their genetic makeup [4,6,8,10].

Among the recognized genetic risk factors associated with thrombophilia, *SERPINE1* elevated gene expression has been identified as the most robust in pediatric populations affected by thrombosis [9]. PAI-1, mainly released by endothelial cells, is the predominant inhibitor of fibrinolysis, since its binding to its antagonist, tPA, thus blocking the conversion of plasminogen to active plasmin, which in turn normally degrades fibrin, a proteinic polymer that stabilizes the thrombus [2,13,18,19]. Higher levels of PAI-1 lead to hypercoagulability by diminishing fibrinolysis, thus increasing the risk for thrombus development, while thrombin itself stimulates additional PAI-1 release in a coagulation-enhancing feedback loop [13,15,16,17,20]. PAI-1 levels demonstrate a progressive rise a priori to the thrombotic incident, peak during the acute phase, and thereafter decline after the completion of the administered treatment regimen that corresponds to successful thrombus-resolution and additional prophylaxis [5,15,22,23]. Fibrinolytic capacity of more than 50% of patients remains impaired for the long term, for at least 3 months after the thrombotic event, due to PAI-1/t-PA disproportion, which can be attributed to prolonged *PAI*-*1* upregulation [24].

The functional 4G/5G promoter polymorphism of the *SERPINE1* gene is strongly associated with increased circulating PAI-1 levels, leading to subsequent hypofibrinolysis and hypercoagulation, in light of the 4G allele, and has been recently demonstrated as the most common predisposing variant in childhood thrombosis, which can additionally exacerbate already impaired childhood fibrinolysis [9,25,26]. Further to the functional genetic variation in *SERPINE1* itself, its levels are positively mediated by ACE, through its active proteolytic product angII, which triggers PAI-1 secretion, therefore inhibiting fibrinolysis and resulting in hypercoagulability [27,28,29]. The functional I/D polymorphism of the *ACE* gene that elevates its expression (peaking at D homozygosity) and activity has been strongly correlated to significant PAI-1 increase, through the ACE/PAI-1 axis [30,31,32].

The implication of miRNAs in adult thrombosis is being actively explored, with mounting and highly promising results [33]. However, their role in pediatric thrombosis remains completely uninvestigated, with no relevant studies published as of yet, possibly due to the rarity of the condition, which restricts sample collection and subsequent research. Therefore, until now, pediatric thrombosis has demonstrated a vast research gap in miRNA epigenetic involvement. This is intensified since corresponding data from adult thrombosis cannot be reliably merged or extrapolated due to the fundamentally different pathophysiology of the two entities, given the central role of impaired fibrinolysis in the pediatric form, a relevant and consistent biological phenomenon from neonates to adolescents.

In the present study, we evaluated serum expression levels of miR-145-5p and miR-34a-5p miRNAs in pediatric patients aged between 0.6 and 16 years who had undergone thrombosis over the past 1–18 months (Table 1, Figure 1). Within the patient cohort, we assessed the expression profiles across distinct post-thrombotic time groups and in relation to treatment status, including recent completion of treatment or administration of chemoprophylaxis. Potential associations between miRNA expression and the patients’ genotypes for the *SERPINE1*-4G/5G and *ACE*-I/D functional polymorphisms were additionally explored. The expressional patterns of miR-34a were further evaluated in relation to family history for thrombophilia, anatomical site of thrombosis, and type of onset (provoked/unprovoked).

Both studied miRNAs were found to hold conserved binding sites in the 3′-UTR of the *SERPINE1* gene with perfect complementarity and have been experimentally shown to negatively regulate its expression through direct experiments. Between these two *SERPINE1* regulators, miR-34a was bioinformatically shown to form an even more stable complex and also appeared to target the mRNA of the *ACE* gene and negatively regulate its expression. Lastly, miR-34a was predicted to strongly target the mRNA of the *SERPINF2* gene, which codes for α2-antiplasmin, the main inactivator of fibrinolytic plasmin [38,39] (Figure 1).

Following RNA extraction and reverse-transcription, the levels of miR-145 and miR-34a were quantified by real-time qPCR through absolute quantification (copies/μL), in order to avoid bias induced from reference-gene instability across different developmental stages of the incorporated age groups. The guiding hypothesis for the present research was based on an anticipated regulatory interplay between the levels of miR-145 and miR-34a expression and PAI-1 fluctuations, as documented in the literature, across different stages of the thrombotic course, clinical parameters, but also different genotypes of PAI-1-increasing functional genetic variants.

**Figure 10:**
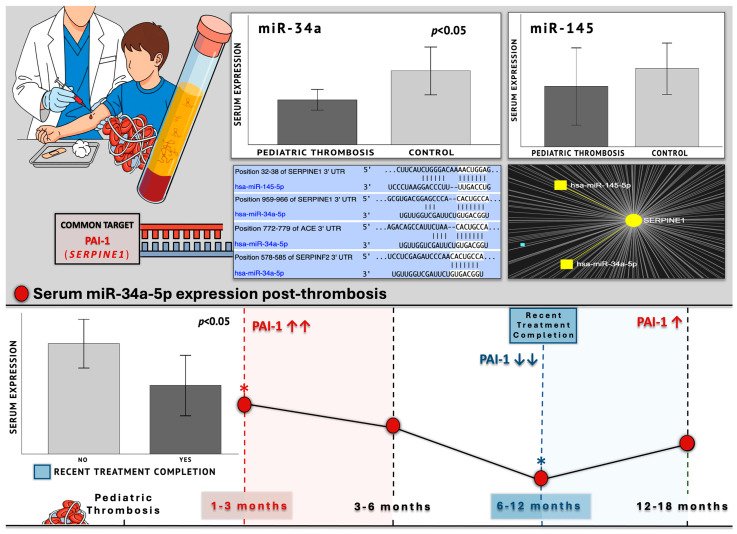
Graphical abstract of study design and key findings upon miR-34a expression in pediatric post-thrombotic course, as well as visualized outputs from target-prediction in silico analyses (TargetscanHuman, miRNet) presented alongside experimental results. Bioinformatic analyses revealed. Target-prediction analyses yielded that miR-145-5p and miR-34a-5p hold conserved binding sites on the 3′-UTR of *SERPINE1* (*PAI*-*1*) mRNA, both scoring perfect complementarity of the miRNA seed region, while in the case of miR-34a, mRNA binding exhibits possibly increased stability due to the presence of an adenine, opposite to miRNA position 1 of the seed sequence. MiR-34a additionally holds 7/7 binding sites on the 3′ UTR of the mRNA of the *ACE* gene, which codes for the upstream positive regulator of PAI-1, as well as on the mRNA of the *SERPINF2* gene that codes for the anti-fibrinolytic α2-antiplasmin. The expression of miR-34a was significantly decreased in patients compared to controls, while no difference was observed for the levels of miR-145 between the two studied cohorts. The lower panel presents a combinatory graph that illustrates the expressional trajectory of miR-34a across the post-thrombotic course with a peak at 1–3 months post-thrombosis, a time when PAI-1 is anticipated to be significantly increased. Its expression follows a gradual decline and finally a *nadir*, shortly after treatment completion, when PAI-1 has been demonstrated to diminish. At the long-term post-thrombotic stage of 12–18 months, where *PAI* expression is expected to recover, miR-34a shows a slight, non-significant increase reaching lower levels than those of the studied controls. These findings are indicative of a dynamic yet impaired regulatory relationship between miR-34a and *SERPINE1*, as well as its upstream controller *ACE*. Hence, the downregulation of miR-34a in pediatric thrombosis might possibly lead to deficient fibrinolytic capacity through the inadequate suppression of the anti-fibrinolytic ACE/PAI-1 axis, the upregulation of which is critical for thrombosis development. Arrows are used for the indication of PAI-1 fluctuations at certain points of the post-thrombotic course, representing upregulation (upward arrow) and downregulation (downward arrow), while asterisks denote statistically significant differences in miR-34a expression between post-thrombotic time intervals.

Our findings suggest that the expression of serum miR-34a was significantly reduced by approximately 40% in the patient group compared to the studied healthy children (FC = 0.61; *p* = 0.029), while miR-145 did differ significantly between patients and controls (*p* = 0.068). In regard to different post-thrombotic time groups (Table 1), the 1–3- and 6–12-month groups noted a significant difference, with the latter exhibiting 45% reduced expression of miR-34a (*p* = 0.031). Nevertheless, the visual inspection of the overall miR-34a expression patterns suggested a characteristic trajectory (Figure 10). The highest levels within patients were observed during the 1–3-month period, which is the shortest after thrombosis, and *SERPINE1* expression is expected around its peak. Followingly, a gradual decline of miR-34a levels was observed, which ultimately reached its lowest point at 6–12 months. This expressional *nadir* coincided with the recent completion of treatment or chemoprophylaxis (1–3 months) within this specific patient subpopulation, therefore aligning with the anticipated diminishing in PAI-1 levels. Thereafter, reaching the 12–18-month group, the expression of miR-34a demonstrated a subtle increase, which was still 39% lower than its respective expression in the control population (Figure 1). Indeed, the recent completion of treatment induced a significant (38%) decrease in miR-34a levels (*p* = 0.048), thus suggesting a temporarily dependent effect, possibly reflecting an absence of necessity for *PAI*-*1* regulation, owing to its typically diminished state following a successful treatment course [5,15,22,23].

The expression of miR-34a was highly influenced by the anatomical site of the developed thrombi (*p* = 0.019), with a peak at cerebral thrombosis, which noted a 76% increase compared to the expressional *nadir* of pulmonary embolism (*p* = 0.007). The synergistic interaction of cerebral involvement and disease-onset type (provoked/unprovoked) was also shown to significantly influence miR-34a levels, with injury-induced cerebral strokes demonstrating a notable increase (37%) when compared to the respective cerebral unprovoked incidents, thus indicating that thrombosis onset type exerts differential effects on miRNA expression, depending on the affected organ.

The levels of miR-34a did not significantly differ in light of the different genotypes of the SERPINE1-4G/5G and/or ACE-I/D functional polymorphisms. However, a clear tendency for upregulation was observed in the levels of miR-34a in the genotypic presence of the 4G and D alleles, which are known to ultimately increase PAI-1 levels. Although this increasing trend of miR-34a is biologically plausible, it is possible that statistical significance might have been influenced by imbalanced genotypic distributions, since 84% of patients carried the 4G allele of *SERPINE1* and 95% carried the D allele of *ACE*.

The actual influence of the studied genetic variables was later revealed through two significant findings, which highlighted the underlying genetic/epigenetic and environmental interactions. The expression miR-34a appeared to be highly influenced by thrombosis site and onset-type, under the presence of the D allele (I/D and D/D genotypes), with cerebral trauma-induced cases that carried at least on D allele, demonstrating an almost 50% increase in miR-34a expression, compared to unprovoked cerebral ones and a 5.2-fold increase, compared to unprovoked non-cerebral thrombotic events (*p* = 0.035).

**Figure 11:**
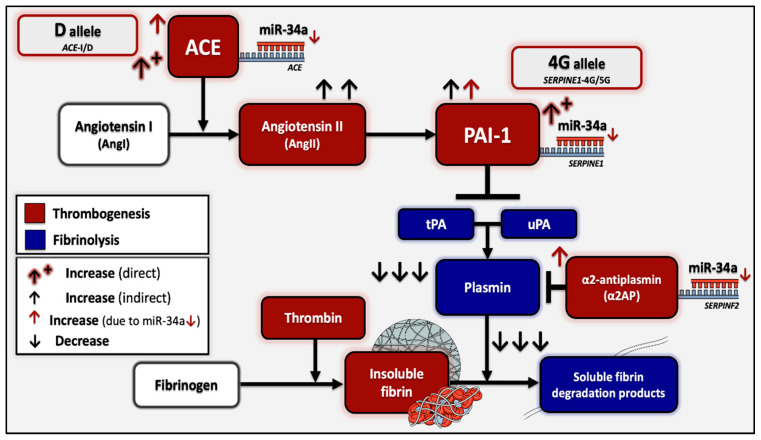
Schematic overview of the interacting mechanisms of thrombogenesis and fibrinolysis, as well as the negative regulation of fibrinolysis through the ACE/PAI-1 axis and α2-antiplasmin. Those illustrated in red are the compounds that promote thrombosis and in blue are depicted the agents that act in favor of fibrinolysis. The diagram additionally illustrates the effect of the functional *SERPINE1*-4G/5G and *ACE*-I/D polymorphisms, which are known to increase the levels of the respective encoded factors and also exhibit a synergistic effect in the upregulation of PAI-1 levels, thus resulting in fibrinolytic decline. The figure further summarizes the targeting of *ACE*, *SERPINE1*, and *SERPINF2* genes by miR-34a, as well as the anticipated effects of its observed downregulation, on fibrinolytic capacity.

Taken together, the above observations are highly consistent with a regulatory role, whereby miR-34a acts in a compensational manner to reactively counterbalance the quantitative rise in its targets (*SERPINE1*, *ACE*, and possibly *SERPINF2*) (Figure 11). Its significant downregulation—by almost half—in the patient population compared to controls, which persists even 12–18 months post-incident and prolonged treatment discontinuation, reflects the long-term impairment of its regulatory capacity. Further support to this conclusion comes from miRNA studies in adult thrombosis, placing miR-34a among the reactively upregulated molecules during the acute phase of thrombotic events, such as ischemic stroke [40,41], where PAI-1 is known to steeply rise [5,15,22,23].

The robust effect of injury-induced cerebral involvement is completely in line with both the established upregulation of miR-34a during the acute phase of adult ischemic stroke, but also with its functionally demonstrated role to epigenetically counterbalance injury-induced inflammation, thus acting as a protective mechanism [42,43]. In fact, not only is it strongly involved in the acute response to mechanical injury, consistent with the provoked pediatric stroke cases we analyzed, but also its higher serum levels over time have been reportedly associated with successful remission in cases with spinal cord injury. The overall serum levels of injured patients were significantly downregulated compared to non-injured individuals, in accordance with our thrombosis-related findings over time, while their assessment has been suggested as a monitoring approach for spinal injury status [44].

These observations agree with the expressional serum-level trajectory of miR-34a that was detected in our study, with its highest levels observed in the time period closest to the thrombotic event, reflecting a compensatory response, followed by its sharp, significant downregulation with treatment completion, in the setting of pharmacologically induced remission, where thrombosis-related target pathways are externally suppressed. Finally, its tendency to increase in light of the long-term absence of treatment highlights a partial yet inadequate recovery that confirms its normally protective pro-fibrinolytic role. However, the expression decrease remains both in the initial reactive response but also long-term after thrombosis, when compared to healthy children with expectedly normal miR-34a function and more efficient hemostatic mechanisms. The latter strongly points out the substantial inadequacy of the miRNA to normally function as an anti-thrombotic protective mechanism, through the effective quantitative regulation of the key pediatric thrombosis factor PAI-1 and its upstream activator, ACE, in the context of the anti-fibrinolytic ACE/PAI-1 axis, but also α2-antiplasmin that normally contributes to hypofibrinolysis [28,39].

This conclusion gains further strength as the overwhelming majority of studied patients (84%) carried at least one *SERPINE1* 4G allele, which directly increases the mainly implicated *SERPINE1* gene at all times, while nearly all patients (95%) harbored at least one *ACE* D allele that has been renown for significantly increasing both levels and activity of its positive regulator and results in even greater levels of pro-thrombotic PAI-1 [26,32]. Therefore, the interpretation of findings cannot be solely attributed to epigenetic regulation rather than a strong interaction between genetics, epigenetics, and environmental factors, in complete accordance with the multifactorial nature of thrombosis.

This notion was additionally confirmed by the detection of an epistatic interaction between the *SERPINE1* and *ACE* genes that was observed to highly influence the abundance of miR-34a in cerebral thrombotic incidents (*p* = 0.006). More specifically, the lowest serum miR-34a levels were observed under the state of double homozygosity (4G/4G + D/D) for the *SERPINE1*-4G/5G and *ACE*-I/D functional variants. This pattern was completely reversed in 4G/4G carriers without concurrent D homozygosity, which appears to act as the genetic switch. This leads to the conclusion that, probably, in light of double 4G4G + D/D homozygosity, where the maximum possible resultant levels of PAI-1 are secreted, the already impaired miR-34a, although not quantitatively enough, is forced to regulate both increased targets that synergistically lead to PAI-1 overflooding. This inadequate response allows for ultimate fibrinolytic dysregulation in children with genetically determined thrombophilia, which, when combined with their immature hemostatic system, can pave the way for thrombosis development.

This might provide a combinatorial genetic/epigenetic predisposing profile, which is highly possible to account for pediatric thrombosis development itself, by acting as a multivariate molecular switch in light of an environmental trigger, such as injury. This conclusion is furtherly supported by the fact that *SERPINE1*-4G/5G polymorphism has been recognized as the predominant risk factor for childhood thrombophilia, among many established risk factors that are considered much more important in adult thrombosis [9]. Since, to our knowledge, the present exploratory piece of research is the first conducted in the context of pediatric thrombosis and miRNA profiling so far, we anticipate that it will lay the groundwork for the future broadening of predisposition from a solely genetic term to a genetic/epigenetic dynamic concept.

### Study Limitations

A limitation of the present study is the relatively small sample size, largely attributable to the rarity of cases, which may adversely affect statistical power, thereby partly elucidating the lack of significance in certain overall comparisons, despite consistent patterns and significant pairwise differences (*p* < 0.05). Moreover, the wide dispersion in the expression values, commonly reported in miRNA studies, further contributes to this matter, thus possibly reducing the power of omnibus tests (one-way ANOVA, Kruskal–Wallis, and Jonckheere–Terpstra). Therefore, biological plausibility should be cautiously co-evaluated in light of results demonstrating contradiction between statistical significance and clearly observed trends, before definite conclusions are drawn.

The present study holds 85.1% statistical power to detect large effects, rather than small or incidental variations. However, power can be reduced in subgroup analyses due to even smaller cohorts, where non-borderline *p*-values and biologically plausible results minimize the likelihood of spurious findings and support robustness of the associations, despite limited power.

## 4. Materials and Methods

### 4.1. Sample Collection

This study was bioethically approved by the Ethics Committee of the Scientific Council of “Aghia Sophia” Children’s University Hospital (7516/01-04-19). Whole blood samples of 38 children with an age range of 0.6–16 years (19 patients and 19 gender and age-matched healthy controls) were collected at the Haemophilia Center for Children and Adolescents/Haemostasis and Thrombosis Unit of “Aghia Sophia” Children’s University Hospital. Eligible participants were neonates, children, and adolescents with radiologically confirmed thrombotic episodes who had been hospitalized for this particular reason. Patients with systemic comorbidities that have been demonstrated to induce thrombosis, such as malignancy and congenital heart disease, were excluded. The control group consisted of healthy neonates, children, and adolescents without any history of thrombotic incidents.

Patients were recruited during post-thrombosis routine follow-up visits. After thorough informative discussion, the parents of the enrolled participants provided written consent for their offspring’s blood samples to be collected and processed for subsequent molecular analyses, as well as for selected clinical data to be co-evaluated. In addition, a thorough four-generation family history of the patients was obtained. Blood samples that were intended for serum separation were drawn directly into collection tubes containing clot-activator, while samples intended for genomic analysis were collected in EDTA-containing tubes.

### 4.2. Serum Separation and RNA Extraction

Whole blood samples with clot-activator remained at room temperature for 15–30 min, to allow clotting to take place. The samples were subsequently centrifuged at 3000 rpm, at 4 °C for 10 min, using a swing-bucket rotor centrifuge. The supernatant serum phase was carefully collected and centrifuged again for additional sample purity.

RNA extraction was performed using the “NucleoSpin miRNA Plasma, Mini kit for circulating miRNA” (Macherey-Nagel GmbH, Dueren, Germany), according to the manufacturer’s protocol for serum samples and RNA elution in the minimum recommended volume, to obtain maximum miRNA concentrations. The concentration (ng/μL) and purity of the eluted RNA were assessed using a BioSpec-nano Spectrophotometer for Life Science (Shimadzu Corporation, Kyoto, Japan). RNA was reverse-transcribed into cDNA directly after extraction, quantification, and normalization across sample concentrations to ensure template comparability.

### 4.3. Bioinformatic Target Prediction Analyses

Target prediction and retrieval of experimentally validated miRNA/mRNA interactions were combinatorially performed via the utilization of TargetScanHuman, miRNet 2.0, as well as TarBase.v.9 (incorporating DIANA-microT-CDS) computational algorithms. Since pediatric thrombosis is mainly driven by hypofibrinolysis, we focused on identifying miRNAs that might contribute to this condition through their expression dysregulation. Hence, the selection started by targeting the mRNA of *SERPINE1*, which codes for the main inhibitor of fibrinolysis, PAI-1, the key player in the pediatric setting. Only miRNA candidates with perfect seed complementarity (7/7 nucleotides) and evolutionary conserved 3′-UTR binding sites were considered for subsequent analysis, and only those with an experimentally validated regulatory relationship with *SERPINE1* were finally considered (>5 functional experiments, including direct validation). Additional priority was given to additional conserved (7/7) targeting and validated regulation of the *ACE* mRNA, given the regulatory role of ACE in PAI-1 levels. To ensure high-confidence miRNA/mRNA complexes, only interactions presenting with microT scores >0.7 were considered. Finally, selection was performed among miRNAs that have been demonstrated to be implicated in adult thrombosis, where impaired fibrinolysis (although not the central mechanism compared to hypercoagulability) also plays a crucial role. On that basis, miR-145 and miR-34a were selected, as both target and have been experimentally shown to regulate the expression of *SERPINE1*, with miR-34a additionally acting as a negative regulator of *ACE*, but also targeting the mRNA of *SERPINF2,* which codes for another crucial anti-fibrinolytic agent (α2-antiplasmin) [38,39].

### 4.4. Reverse Transcription and Quantitative Real-Time PCR

Reverse Transcription (RT) of miRNAs was performed using the miRCURY LNA RT Kit (Qiagen, Hilden, Germany) for miRNA polyadenylation and first-strand cDNA synthesis in a single-tube reaction of 10 μL (2 μL 5xRT SYBR-Green Reaction Buffer, 2 μL RNA template, 1 μL 10xRT Enzyme-Mix, and 5 μL RNase-free water). The RT reaction was performed using a Gradient Thermal Cycler (Takara Bio, Shiga, Japan) and involved incubation at 40 °C for 60 min, an inactivation step at 95 °C for 5 min, followed by subsequent cooling of the reaction at 4 °C. The cDNA samples were stored at −20 °C overnight and processed the following day.

Since the studied cohorts included children from an age range of 0.6–16 years (Table 1), which corresponds to various developmental stages, the use of a conventional reference gene for relative quantification was deemed unreliable due to possible lack of stability. Therefore, absolute quantification of the expression (copies/μL) of the two studied miRNAs (miRBase accession numbers: MIMAT0000255 and MIMAT0000437) was performed using standard curves, produced from serial 2-fold dilutions of reverse-transcribed synthetic RNA oligonucleotides corresponding to the mature miRNAs (including matching length, GC content, and molecular weight). The produced curves demonstrated high linearity (R^2^ ≈ 0.99), and efficacy within the acceptable range of 90–110%. As an illustrative example, for miR-34a, the efficacy was 96%, with an R^2^ of 0.9915. Reverse transcription of the synthetic RNA molecules was performed under the same protocol as the target serum-miRNAs, which were analyzed in this study (LNA series, Qiagen, Hilden, Germany).

Quantification was conducted by SYBR^®^ Green-based, real-time quantitative PCR (qPCR), performed on a LightCycler 480ii real-time PCR instrument (Roche, Basel, Switzerland). Each reaction of 10 μL contained 5 μL of 2XmiRCURY SYBR-Green Master Mix, 4 μL cDNA, and 1 μL miRCURY LNA miRNA PCR Assay (Qiagen, Hilden, Germany) for the respective miRNA. The qPCR protocol involved heat activation at 95 °C for 10 min, followed by 45 cycles of 95 °C for 15 s and 60 °C for 1 min, and finally a melting-curve analysis cycle ranging from 60 °C to 95 °C, with a ramp rate of 0.03 °C/s and continuous fluorescence acquisition.

Threshold cycles (Ct values) were obtained through the instrument’s software by employing the “Absolute Quantification/2nd Derivative Max” analysis. Although the experimental protocol incorporated 45 cycles of amplification, a threshold of 40 cycles was set for reliable detection.

### 4.5. PAI-1 Genotyping for the PAI-1 (SERPINE1) 4G/5G Polymorphism

Genomic DNA was extracted from white blood cells of the patients’ EDTA-containing whole blood samples, using the NucleoSpin Blood kit (MACHEREY-NAGEL, Germany). Genotyping of the *PAI*-*1* 4G/5G polymorphism was conducted by PCR amplification, performed on a Gradient Thermal Cycler (Takara Bio, Japan), using the following primers: 5′-CACAGAGAGAGTCTGGCCACGT-3′ and R: 5′-CCAACAGAGGACTCTTGGTCT-3′. Thermocycling included initial denaturation at 94 °C for 4 min, followed by 35 cycles consisting of a denaturation step at 94 °C for 0.5 min, subsequent annealing at 57 °C for 1 min, and an elongation step at 72 °C for 1 min. The final 5 min elongation step was set at 72 °C, ultimately resulting in an amplified fragment of 99 bp [45].

The obtained PCR products underwent overnight incubation with the BslI restriction enzyme at 37 °C, which resulted in a 77 bp and a 22 bp fragment in the presence of the 5G allele. Genotyping of the studied samples was carried out through the visualization of the DNA fragments under UV-light, following agarose gel electrophoresis and nucleic acid staining with GelRed fluorescent stain (Biotium, Fremont, CA, USA).

### 4.6. ACE Genotyping for the ACE I/D Polymorphism

Genotyping of the *ACE* I/D polymorphism was conducted by PCR amplification, performed on a Gradient Thermal Cycler (Takara Bio, Japan), using the following primers for I or D allele amplification: F: 5′-CTGGAGACCACTCCCATCCTTTCT-3′ and R: 5′-GATGTGGCCATCACATTCGTCAGAT-3′.

The thermocycling protocol included initial denaturation at 94 °C for 4 min, followed by 35 cycles consisting of a denaturation step at 94 °C for 0.5 min, subsequent annealing at 60 °C for 1 min, and a 1 min elongation step at 72 °C. The final 5 min elongation step was set at 72 °C. The PCR amplification resulted in two DNA fragments of 490 and 190 bp, respectively, corresponding to the I and D alleles [45]. Genotyping was performed under UV light, following agarose gel electrophoresis and nucleic acid staining.

### 4.7. Statistical Analysis

The total of statistical analyses was performed using IBM SPSS Statistics, Version 29.0.2.0. Normality was assessed using the Shapiro–Wilk test for continuous values, according to sample size. Two-sample comparisons were conducted by employing the independent-sample *T*-test or Mann–Whitney U test, with respect to the distribution of values. Likewise, comparisons between more than two groups were conducted using “one-way ANOVA” or the Kruskal–Wallis test, as appropriate. Additionally, the Jonckheere–Terpstra test was applied for ordered group comparisons and subsequent pairwise analyses. Finally, to examine the effect of the interaction between time and treatment on miRNA expression, general linear models (GLMs) were employed. Error bars are included in the presented result figures in order to illustrate the variability of observed values around the central tendency (mean). An a priori power analysis (two-sided α = 0.05) indicated that with the included sample sizes (19 patients and 19 controls), the present study had 85.1% power to detect large effects and biologically meaningful differences (Cohen’s d = 1.0). The level of statistical significance was set at *p* < 0.05 (two-tailed).

## 5. Conclusions

Our findings suggest that miR-34a-5p might normally function as a regulatory “emergency brake” against thrombosis that is driven by PAI-1-induced hypofibrinolysis. Its notable long-term decrease in pediatric patients with thrombotic incidents provides a mechanistic hint that failing to adequately regulate the expression of the *SERPINE1* and *ACE* genes allows for the unchecked elevation of the ACE/PAI-1 axis, which is already renowned for its implication in thrombosis development through the diminishing of fibrinolysis. MiR-34a constitutes a promising biomarker for real-time assessment of fibrinolytic capacity in children at high risk for thrombosis due to genetic and/or environmental factors, or in pediatric patients following a thrombotic incident, in order to assess recurrence risk. Further research in larger cohorts, as well as functional studies, is deemed necessary in order to reveal its exact role in hemostasis and to establish its potential utility in the clinical setting.

## Figures and Tables

**Table 1:** Characteristics and clinical data of the studied patient and control groups. Variables such as age and gender were statistically compared between the two groups by utilization of the appropriate statistical tests. The level of statistical significance was set at *p* < 0.05. The groups studied did not differ significantly as to the variables of age and gender. ^1^ Mean (Range); ^a^ Mann–Whitney U test (2-sided), ^b^ Pearson Chi-Square (2-sided), ^c^ Independent Samples *T*-test.

Variable	Patients (*n* = 19)		Controls (*n* = 19)		*p*-Value
**Age** (years) ^1^	9.51 (0.6–16)		7.03 (0.6–15)		0.124 ^a^
**Gender** *n* (%)	**Male**	**Female**	**Male**	**Female**	0.163 ^b^
	15 (78.9%)	4 (21.1%)	11 (57.9%)	8 (42.1%)	
**Thrombosis Patients** (*n* = 19)					
**Type of Thrombosis** (*n*, %)					
Arterial (*AT*)			Venous (*VTE*)		
1 (5.3%)			18 (94.7%)		
**Anatomical Site of Thrombosis** (*n*, %)					
Cerebral		Lower Limbs		Pulmonary	
9 (47.4%)		6 (31.6%)		4 (21.0%)	
**Thrombotic Incident Classification** (*n*, %)					
Provoked			Unprovoked		
6 (31.6%)			13 (68.4%)		
**Months post-Thrombosis ^1^**					
7.63 (1–18)					
**Time Groups** (months; *n*, %)	**1–3**	**3.1–6**	**6.1–12**		**12.1–18**
	4 (21.1%)	5 (26.32%)	7 (36.8%)		3 (15.79%)
**Treatment**	Yes (47.37%)		No (52.63%)		
**Family History of Thrombosis** (*n*, %)					***p*-value**
	Positive		Negative		
	12 (63.2%)		7 (36.8%)		0.710 ^c^
***PAI*-*1* (rs1799889) Genotype** (*n*, %)					
**5G/5G**		**4G/5G**		**4G/4G**	
3 (15.8%)		12 (63.2%)		4 (21.1%)	
***ACE* I/D (rs1799752) Genotype** (*n*, %)					
**I/I**		**I/D**		**D/D**	
1 (5.3%)		11 (57.9%)		7 (36.8%)	

## Data Availability

Original data presented in this research shall be available upon request to the corresponding author. Certain data may be subject to limited availability in accordance with confidentiality and privacy protecting protocols or temporary ongoing study conduction.
